# Postural Analysis in Ventral and Dorsal Decubitus Babies Using Deep Learning Techniques: A Protocol Study

**DOI:** 10.3390/jcm14093096

**Published:** 2025-04-30

**Authors:** Sara Velázquez-Iglesias, Vidal Moreno-Rodilla, Belén Curto-Diego, Fátima Pérez-Robledo, Rocío Llamas-Ramos, Jose Ignacio Calvo-Arenillas, Inés Llamas-Ramos

**Affiliations:** 1 Department of Nursing and Physiotherapy, Universidad de Salamanca, 37007 Salamanca, Spain; 2 G.I.R “Robotics and Society”, Dpt. Computers and Automation, Faculty of Sciences, Universidad de Salamanca, 37008 Salamanca, Spain; 3 Instituto de Investigación Biomédica de Salamanca (IBSAL), 37007 Salamanca, Spain; 4 University Hospital of Salamanca, Health Service of Castile and Leon (SACyL), P.º de San Vicente, 182, 37007 Salamanca, Spain

**Keywords:** artificial intelligence, deep learning, motor development, neural networks, postural analysis

## Abstract

**Background:** The analysis of posture in the early stages of motor development has always been a subject of research and study. With the evolution of new technologies, the need arises to implement evaluation tools that allow an objective and effective assessment of postural control, which is intrinsically linked to motor development. **Objectives**: The objective was to analyze posture in babies from 0 to 6 months in ventral and dorsal decubitus using artificial intelligence to determine objective parameters of postural assessment. **Methods**: The study is an observational and cross-sectional study. The babies will be studied following a systematic kinesiological assessment, and the images of the babies will be taken, both in ventral and dorsal decubitus, on a glass platform, to analyze their posture by means of deep learning techniques. **Results**: Many authors have investigated posture in newborns. However, there is no method for assessing motor and postural development to determine the support area of typically developing babies. Artificial intelligence is postulated as an effective tool to objectively analyze the posture of babies and detect possible delays. Using deep learning techniques as a predictive tool, the support areas of each baby will be defined according to their age. **Conclusions**: Early detection of motor or postural developmental delays in babies to optimize effective treatment is of great importance. Artificial intelligence can help manage the complexity and growing volume of data in healthcare by knowing the correct postural control at each stage of a baby’s early months, while reducing the workload of healthcare professionals by facilitating decision-making.

## 1. Introduction

The human genus cannot be conceived without posture and motricity. That is why the significance of postural control in humans is extremely relevant, since it is the result of innumerable phylogenetic adaptations [1].

Postural control is closely linked to motor development in humans because it is a fundamental part of postural control and movement coordination. Postural analysis in early developmental stages describes the evaluation and study of the baby’s body posture to identify and analyze possible deviations, irregularities, compensations, or alterations in the alignment of the different structures of the body, such as the head, spine, and limbs. The development of gross motor skills (such as sitting, standing, crawling, or walking) depends fundamentally on postural control. The basic structural commands for postural control are present shortly after full-term age, but it takes many years to reach adult postural control configuration [2].

After an uncomplicated delivery, the term newborn has an innate program for interacting with the environment. The baby has a certain automatic postural control, which is not only an indispensable condition for any movement, but both processes are closely overlapped during the whole motor action [3]. This postural analysis, in the early stages of motor development, has always been the subject of research and study over the years. In the first two trimesters of life, the postural control of babies in dorsal and ventral decubitus is in continuous progress towards stability, which is why it is fundamental in the development of the motor milestones that the baby will reach throughout his or her life.

The kinesiological assessment of posture can be based on the analysis of postural ontogenesis, which comprises the innate motor and postural patterns that develop in the first year of life of the baby [4]. The study of ontogenesis differentiates between patterns that develop from the ventral decubitus and those described from the dorsal decubitus. The ventral decubitus is analyzed to evaluate motor patterns involved in the functions of straightening and support against gravity, and the dorsal decubitus is studied to evaluate motor patterns related to the function of grasping [5].

At present, among the posture analysis tools are deep learning (DL) techniques, a subset of machine learning (ML), which is one of the most active branches in the field of artificial intelligence (AI). More specifically, these DL techniques are based on Artificial Neural Networks (ANNs) with multiple layers (hence the term “deep”) to generate models from previous input data, without the need to specifically program those models, which will be used to make decisions on new input data. There are several architectures and types of Deep Neural Networks (DNNs), each designed for different tasks, such as Convolutional Neural Networks (CNNs) that are specialized for processing data with a grid-like structure, like images or video. CNNs can detect hierarchical features and patterns within images and videos, so they have direct application in specific tasks such as object detection, image classification, facial recognition, and semantic segmentation, among others. Within the field of image processing, this concept of semantic segmentation refers to the ability to define different areas in images (segmenting) so that they have a meaning or semantics. Unlike image classification, which assigns a single label to the entire image, semantic segmentation identifies which area of the image belongs to which class. In an image of an urban scene, a CNN for semantic segmentation would divide the pixels in the image into several classes, such as car, road, pedestrian, etc. The output of the segmentation network would be a segmented image where each pixel belongs to one of these classes, effectively creating a mask for each class in the image [6].

DL, and more specifically CNNs, therefore play a vital role in the field of computer vision and, within it, in image analysis with applications in multiple areas, such as medical image analysis. The purpose of DL is to replicate capabilities such as semantic segmentation, as well as image classification. To build a model that replicates these capabilities, one must initially have a dataset consisting of multiple pairs of original and labelled images, covering the complete range of cases that the CNN must learn. A neural network can be thought of as a distributed computational system that implements various mathematical models, which must be discovered and defined without human intervention, yet it is able to perform the same tasks as a human or specialist. The parameters of these models, called adaptive because they “adapt” to specific problems, are tuned in what is defined as a learning stage using a Training Set. The Training Set is a subset, typically 80%, of the dataset, containing multiple pairs, where each pair consists of the original image and the corresponding human-labelled image. Using these data, the process known as learning or training takes place, during which the network learns to perform a specific task, such as image classification or segmentation. During this process, the network adjusts its internal parameters (weights and biases) to minimize the error between the network’s outputs and the expected true values (or labels). The Training Set is image and interpreted image pairs. Usually, it is necessary for an expert to use this Training Set to define and establish the facts and their interpretation manually. With this information, what is called the learning or training process is carried out. Likewise, a validation process must be defined to ensure the network’s performance on images not used (Validation Set) during the learning phase, i.e., to confirm that the neural network can generalize [7].

As expected, the dataset must meet certain key characteristics to ensure that the model can learn effectively and generalize well to new data or images. These characteristics include a variety of representative and balanced problem cases, different lighting conditions, noise, and object variations to help the network generalize better, as well as high-quality labels and annotations with minimal errors. There are several already-built datasets that are widely used to train CNNs in various image analysis tasks. Some of the most popular include ImageNet, CIFAR-10, CIFAR-100, and MNIST for image classification tasks, and COCO, ADE20K, and CamVid for semantic segmentation. These datasets are a great starting point for creating and evaluating CNNs, whether training a model from scratch. However, when fine-tuning a pre-trained neural network for a particular application, it is highly beneficial to build a specific dataset with images that are representative of the domain in which the network will primarily operate. In this way, the trained model will be more accurate and provide better performance on the task at hand, improving its generalization capacity.

Considering the enormous potential offered by the DL technologies available within the field of computer vision in medicine, we intend to develop an evaluation system to assist the specialist in the analysis of babies’ posture. The system will enable an objective and efficient assessment of posture to evaluate the motor development of newborns, as well as to identify possible problems that may require early intervention. The evaluation system will rely on real-time image processing, extracted from motion videos of babies. Using a deep learning approach based on semantic segmentation, the areas of support in babies’ dorsal and ventral decubitus positions will be detected. Once the areas of support (key features) are identified, automatic reasoning techniques will be employed to generate objective parameters that help systematically detect postural disorders.

Central coordination is of vital importance to palliate or considerably reduce the progress or appearance of a delay in motor development, hence the enormous importance of research in early detection, in the first months of life of the infant. Therefore, having objective parameters to determine neurodevelopmental delays would provide health professionals with a tool of great predictive value.

This paper presents a protocol study on a novel and objective approach to real-time analysis of babies’ posture. The document will, therefore, focus on describing the organization, procedure, and materials needed to develop and evaluate the final system.

## 2. Hypothesis and Objectives

### 2.1. Hypothesis

A machine learning algorithm could determine the areas of support in babies with their typical motor development, thanks to prior training.

### 2.2. Objectives

#### 2.2.1. Main Objective

- Employing artificial intelligence to determine objective parameters of postural assessment in babies aged 0 to 6 months.

#### 2.2.2. Secondary Objectives

- To know the support used by healthy, typically developing babies;
- To analyze the base of support in ventral and dorsal decubitus in healthy babies aged 0 to 6 months;
- To determine whether the objective parameters of dorsal and ventral decubitus posture in the first and second trimesters of the baby correspond to the chronological age of each baby previously evaluated;
- To describe an early detection tool that helps to recognize areas of support considered within normality and to differentiate them from different disorders.

## 3. Materials and Methods

### 3.1. Design and Setting

This will be an observational and cross-sectional study whose measurements will be performed uniquely. The babies will be evaluated in a private physiotherapy center. The study has been approved by the Ethics Committee of the University of Salamanca under registration number 840.

The objectives and procedure of the study will be disseminated through health centers, hospitals, and social networks, among others; an informative poster will be produced with contact information to recruit families wishing to participate in the study.

Before the assessment, the parents will be informed about the procedure, the tests to be performed, and the absence of risks. They will then sign an informed consent form as the child’s legal guardians and be assigned an identification number. The only personal data collected that could identify the baby and its parents is provided in the informed consent form. These data will be stored in a secure file by the IP.

### 3.2. Study Population

The sample will be composed of babies aged 0 to 6 months. Non-probabilistic convenience sampling will be used. Patients were not involved in the design, conduct, reporting, or dissemination plans of this research. Participants will be selected based on the following criteria:

#### 3.2.1. Inclusion

- Healthy babies from 0 to 6 months of age;
- Babies whose parents sign the consent to participate in the study and authorize the taking of images and video.

#### 3.2.2. Exclusion

- Babies older than 6 months of age;
- Babies younger than 37 weeks;
- Babies younger than 6 months of age with described pathology or diagnosed motor retardation.

### 3.3. Variables and Measurement Instruments

The main study variable will be posture, which will be evaluated in dorsal and ventral decubitus using the following instruments.

#### 3.3.1. Medical History

Sociodemographic data of the mother, pregnancy, and delivery will be collected in a Clinical History Sheet. The infant’s sociodemographic data, current situation, and sleeping and eating habits will also be noted.

#### 3.3.2. Postural Support Prototype

A postural support prototype will be designed to assess the baby’s posture in dorsal and ventral decubitus. It will be a device similar to a podoscope, since the ultimate goal is to analyze the support area of children. The prototype consists of a metal structure with a glass top surface on which the babies are placed for image/video acquisition of the support areas in the dorsal and ventral decubitus position. A camera for video recording will be installed on the lower part of the metal structure. The stand must meet a series of design requirements, including safety and comfort for the babies, an adequate ergonomic posture for the professionals to perform the assessment, and a defined contour made of a material that avoids reflections. This postural support prototype would serve as a proof of concept to refine the methods, validate the system’s effectiveness, and provide a foundation for future improvements.

#### 3.3.3. Software Environment

Two software tools will be used to build the dataset and carry out the training and evaluation of the CNN: Artstudio Pro and MATLAB.

Artstudio Pro is a software application designed for creating and editing images and digital artwork. It features an intuitive and optimized user interface within the iPad environment with the Apple Pencil, making it an ideal tool for both beginners and more advanced users who wish to work with graphic design tools that support layers. In this work, Artstudio Pro (version 5.2.8) will be used to create a domain-specific dataset for training and evaluating the neural network.

The MATLAB (version 23.2) numerical computation platform incorporates the deep learning Toolbox to develop all learning tasks in the DL workflow, such as the following:

- Preprocess the input images to prepare them for training by resizing them to a uniform size, augmenting the dataset, and splitting the images into Training and Validation Sets.
- Use pre-trained CNNs (in what is usually called transfer learning) or build a new CNN.
- Train the network with configurable options and training loops, to adapt the model parameters to the specific problem.
- Validate the behavior of the network during training.
- Subsequently, evaluate (test) the trained model with images other than those used in training.

Once the network has been trained and validated, MATLAB also offers the possibility of network deployment, in the sense that it allows exporting the network for a specific application and for a processing device (GPU, CPU, etc.). In this way, it can be integrated with other components to build the DL-based system for, for example, real-time image segmentation.

### 3.4. Procedure of Protocol Study

One rehabilitation physician, four physiotherapists, and two computer scientists will participate in the study.

The protocol study procedure for a neural network-based project consists of several stages: completion of the medical history, kinesiological evaluation of the baby, construction of the domain-specific dataset, and the selection, training, validation, and performance evaluation of the neural network for semantic segmentation—typical workflow tasks DL.

#### 3.4.1. Kinesiological Evaluation

Once the medical history sheet has been completed, the rehabilitation physician will perform the kinesiological assessment of the babies. The baby will be naked, will be placed on a stretcher, and while the assessment of the baby is being performed, the data concerning the assessment sheet prepared to facilitate the collection of data by the physiotherapists will be collected.

The kinesiological assessment of motor development will be based on the neurokinesiological diagnosis according to Professor Václav Vojta [5], in which postural and motor development are assessed as part of a global pattern within postural ontogenesis, and based on the level of straightening reached in each motor milestone. In this systematic approach, the spontaneous motor skills of the baby in the ventral decubitus and dorsal decubitus will be assessed first. Subsequently, each of the seven postural reactions will be assessed, which allow the level of straightening to be quantified (reaction to traction, Landau’s reaction, reaction to axillary suspension, reaction to Vojta’s loss of lateral balance, reaction to Collis’ horizontal lateral suspension, reaction to Peipert and Isbert’s vertical suspension, and reaction to Collis’ vertical suspension) and therefore, determine the quality of the baby’s global pattern to relate it to the “ideal pattern”, as maximum attainable postural quality. The difference between both determinants is what results in the “alteration of central coordination”. In addition, the dynamics of the primitive reflexes [5] will be evaluated, relating them to the chronological age of the baby, taking into account the persistence times of each primitive reflex (tonic, osteotendinous, and superficial reflexes).

#### 3.4.2. Image Acquisition Using the Postural Support Prototype

For training the neural network, it is necessary to build an image dataset specifically designed for the analysis of baby posture. To achieve this, a video of the babies in ventral and dorsal decubitus will be taken using the postural support prototype. The lighting of the scene will be taken into account so that there are no shadows, glare, or reflections. The camera that will record the images will be a GoPro, which combines qualities such as high definition, wide angle, and remote control with a mobile device or a computer. One of the physiotherapist researchers will place the baby in ventral and dorsal decubitus. During the time the baby remains on the platform, it will not be offered any type of stimulus to direct its attention, but the baby will be left to move freely so that it can spontaneously regulate its posture on the platform.

#### 3.4.3. Construction of the Domain-Specific Dataset

The objective is to obtain a complete dataset that allows the development of the CNN learning tasks. Once the video has been captured, the frames (frame or specific image within a succession of moving images) will be automatically extracted. These images, along with the corresponding images manually labeled by the physiotherapists with the relevant characteristics, will form the dataset. The manually segmented image (Ground Truth) must be obtained for each frame. For this purpose, the following key features have been defined:

1. Area outside the platform without significant events;
2. Metallic surface of the platform;
3. Internal area of the platform corresponding to the glass;
4. The evaluators and the baby’s parents, if they appear in the image;
5. The baby’s body;
6. The areas of support where the baby makes contact with the glass surface.

The physiotherapist researchers will proceed to colorize or annotate these key features with the Artstudio Pro application in each of the frames. Their expertise in defining the ground-truth dataset is invaluable, making the use of a simple graphical editing tool a key element, especially considering the daunting task of creating a valid dataset. In this way, the real image will be transformed to obtain the corresponding interpreted image (GroundTruth) by a skilled annotator. With the Artstudio Pro, for each of the frames, seven layers will be created using the following color code:

1. Layer 1 will contain the original image as the basis for coloring the other six layers;
2. Layer 2 will contain the entire surface of the image in gray, which will define the ceiling;
3. In layer 3, the outer contour of the glass platform will be delimited and filled in blue color, which will be the metal structure;
4. In layer 4, the inner contour of the glass platform will be delimited and filled in red color, defining the glass surface not occupied by people;
5. In layer 5, the entire surface of the evaluators and the baby’s parents, if they appear in the image, will be marked in orange;
6. In layer 6, the surface of the baby’s body will be marked in yellow color, defining the body of the unsupported baby on the glass surface;
7. In layer 7, the surface of the baby’s support on the glass platform shall be marked in green.

**Figure 1:**
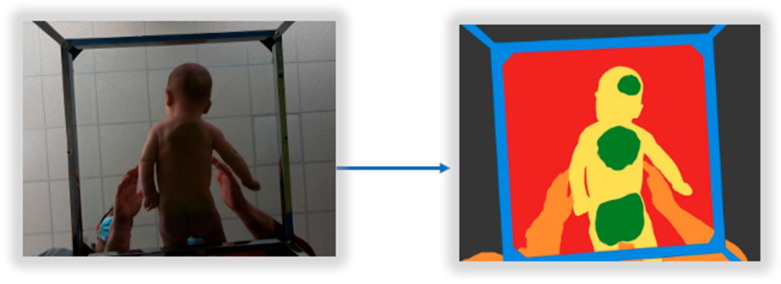
Transformation from real image to dataset image (Ground Truth).

Once all the layers have been superimposed in the indicated order, the resulting image (Figure 1) will contain only significant features; other objects, such as the roof, will be removed, and problems in image processing will be avoided.

#### 3.4.4. Deep Learning Workflow Tasks

Typical neural network training workflow tasks are usually performed using a DL framework, with TensorFlow (by Google) and PyTorch (version 2.7.0.) (by Facebook) being the most popular. In this study, we have chosen to work with the MATLAB development environment (by MathWorks) along with the ‘deep learning’ toolbox, as it integrates programming, visualization, and data analysis in an interactive environment with rapid prototyping capabilities and support for specialized hardware, such as GPUs (Graphics Processing Units). with MathWorks Matlab platform.

With this integrated environment, a CNN will be trained and validated for the semantic segmentation of a new image by assigning a label to each pixel from the predefined set of 6 classes. A CNN has an image as input and generates pixel-level labels through multiple layers of convolution and deconvolution. Over the last few years, various CNN models have been introduced, including VGG, AlexNet, GoogLeNet, and ResNet. Since its introduction in 2015, ResNet50 has revolutionized the field of CNNs by enabling the training of very deep networks without the problem of vanishing gradients [8]. ResNet50 is a 50-layer deep model that addresses this problem through the use of residual blocks, layered blocks that include direct connections between the input and output, which enhance the performance of the CNN compared to other models.

MATLAB includes the pre-trained ResNet50 model, which has been trained on the ImageNet dataset. Training this model with our domain-specific dataset will involve a series of steps, including the following:

- Create two folders: one containing the images extracted from the videos and the other containing the Ground Truth images, which have been tagged by the skilled physiotherapist.
- Define 6 semantic classes, which describe the significant features of the scene: ‘Ceiling’, ‘Metallic-Frame’, ‘Glass’, ‘People’, ‘Unsupported’, ‘Supported’. Each class is assigned an RGB color, respectively: grey, blue, red, orange, yellow, and green.
- Prepare the training and validation datasets. Typically, the training dataset will consist of 80% of the images, while the validation dataset will contain the remaining 20%.
- Load the ResNet50 pre-trained model.
- Configure training options, including the optimizer, momentum value, learning rate, number of epochs, batch size, and other relevant parameters.
- Train the network using the configured options and training data. During training, the pre-trained ResNet50 weights are fine-tuned using backpropagation and the selected optimizer.
- Save the trained model for use in the deployment phase.

As a final result of the training process, C++ code will be generated to run the neural network on a GPU. This will provide the CNN for deployment in the end-user software application.

#### 3.4.5. Statistical Analysis and Performance Evaluation

Previously, the sociodemographic variables of the babies and their mothers were analyzed. The results will be expressed as mean ± standard deviation for quantitative variables and as number and percentage for qualitative variables. Once the descriptive analysis of the variables has been carried out, a correlation analysis will be performed between the variables, as well as a regression analysis to determine if there is any predictive data of the child’s motor development. Linear regression will be performed to determine the predictive value of the variables collected in the functional assessment on the percentage of support surface in infants. Coefficients, confidence intervals, and *p*-values will be calculated for each variable with respect to the support surface. Multivariate regression will also be performed to assess the potential relationship between variables when making predictions about the support surface. The statistical package SPSS v28 software (IBM Corporation, Armonk, NY, USA) will be used.

To describe the progress of the training of the different neural network proposals, the accuracy and loss graphs of the network, which are integrated in the MATLAB environment, will be used. Once the training or learning is finished, the similarity between the segmentation of the Ground Truth images and the predicted segmentation resulting from the trained model will be evaluated. On the areas of the baby rests on the platform glass, the most widespread evaluation metrics in the field of computer vision and in MIS (Medical Image Segmentation) will be used, such as Dice coefficient and Jaccard index (IoU—Intersection over Union), as well as others more widespread in the medical predictive work such as precision, recall (sensitivity), accuracy (rand index), and the confusion matrix.

#### 3.4.6. Objective Parameters in Postural Disorders

Finally, automatic reasoning techniques based on the segmented supports should be developed to describe patterns that show a distance from normality in the kinesiological study, which, given the early stage of the baby’s development, make an adequate treatment feasible. The parameters that might indicate a postural disorder in the baby include the following:

- Area of contact between the baby’s body and the glass surface, e.g., which part of the back or belly is resting on the surface;
- Symmetry, such as checking whether the body parts are positioned symmetrically relative to each other;
- Angles formed between the baby’s body parts, such as the extremities, head, and trunk.

### 3.5. Posture Evaluation System Based on Deep Learning Techniques

**Figure 2:**
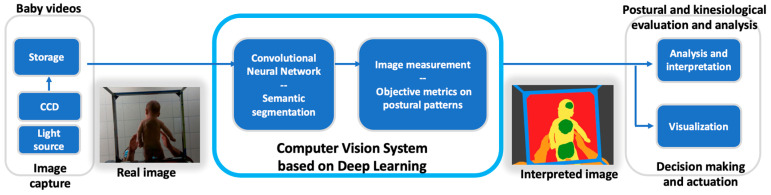
Proposed ML-based machine vision system.

Figure 2 illustrates the proposed system, which is based on deep learning techniques, to assist specialists in assessing a baby’s posture in their daily work. The core of the system will be the CNN, which will result from training and validation with the constructed dataset.

The initial part corresponds to the acquisition of images using a video camera, which is responsible for capturing the frames when the physiotherapist places the new baby on the platform in ventral and dorsal decubitus.

In the center of the figure is the CNN, deployed with MATLAB, for integration into the end-user software application. This CNN will perform the segmentation of these new images, identifying the significant features for which it has been trained, mainly the support of the baby on the glass of the platform.

On the monitor, the physiotherapist will be able to visualize these supports highlighted in green, which will help to identify possible deviations and irregularities in the baby’s structures.

When objective metrics are defined in postural patterns, other AI solutions can be incorporated into the system to classify the images of healthy infants. These objective measures may include the number of supports, the area of the support, and its relationship to the weight and height of the baby, its location relative to the body, and the relationship between the locations of the supports.

## 4. Ethics and Dissemination

The need to systematize in an objective and automatic way the analysis of posture in babies, using AI as a tool due to the fast processing it allows and the systematics it employs in the field of newborn assessments, would speed up developmental assessments in babies, as well as the prevention and detection of delays in motor and postural development. The potential expansion of AI is related to the rapid processing and storage of data, because it enables a wide range of applications in healthcare.

In recent decades, healthcare has been marked by several major events: increasing complexity, growing volumes of data, and burnout of healthcare professionals. Multiple applications of AI could help in solving these problems by generating new knowledge, increasing computational capacity, and reducing the workload of healthcare professionals [9].

Machine learning, as a core DL capability, in pediatrics and neonatology has already been applied and investigated to improve patient care and prognosis, for example, in a study by Matsushita et al. [10] that used machine learning in the identification of different phenotypes in 215 extremely low birth weight babies, given that patient heterogeneity is a cause of failure in clinical trials, machine learning algorithms can find patterns within a heterogeneous group, meaning that not all premature babies should be treated in the same way. Another example is Achenie LEK et al. [11] who used deep learning techniques for the detection of autism in young children, or Le et al. [12] using a machine learning-based algorithm to predict severe pediatric sepsis.

Malerbi et al. [13] commented on the article by Matsushita et al. [9] in which they agreed on the enormous potential of artificial intelligence/deep learning (AI/DL) systems to improve healthcare; however, Malerbi et al. consider that legal regulation of AI/DL, involving system design, validation, implementation and post-market monitoring, is important, while taking measures to avoid biased datasets, thus more research on their safety and efficacy is needed. For risk stratification, regulatory policies could be inspired, for example, by existing data protection legislation. Regulation must balance security guarantees and support for innovations, and must involve all stakeholders to adequately ensure the security, effectiveness, and fairness of the implemented systems. This is a real challenge, which is why there is a proposal for the creation of a new medical specialty of clinical AI that would help clarify all these dilemmas. Collaboration between researchers, developers, ethicists, clinicians, patients, and regulatory agencies is essential for the rapid adoption and successful implementation of AI/DL in healthcare, with enormous potential to optimize care, increase access, decrease cost, and promote equity. Scientific and medical societies should actively participate in the formulation of best practices and the prospective validation of AI systems.

Literature shows that the position of babies has been studied, especially in relation to several aspects. On the one hand, the position that babies acquire when sleeping and motor development [14,15], mainly comparing motor development in babies who sleep in ventral versus dorsal position [15], concluding that babies who sleep in dorsal decubitus may present early motor delays when that time is associated with less time in ventral decubitus when awake, thus concluding that the level of baby motor development is influenced by extrinsic factors such as the baby’s positioning. It is of vital importance to highlight that it has been estimated that the recommendation of sleeping in dorsal decubitus has saved 850 babies annually in Australia and other countries [16]. On the other hand, the relationship between the position of babies while sleeping and their relationship with sudden infant death syndrome (SIDS) [16,17] is being studied. Recently, the literature search conducted by Jullien in 2021 [18] summarized the main findings of systematic reviews related to the prevention of sudden infant death syndrome (SIDS) with the certainty reported evidence in which a decrease in the incidence of SIDS has been observed following the recommendation of “sleeping on the back”, associated with recommendations to sleep in a safe environment that includes a dorsal decubitus position, a firm surface, no soft objects or loose bedding, no head covering, no overheating, and room sharing. Breastfeeding on demand and pacifier use during sleep also protect against SIDS. In addition, research has been performed on the relationship between infant positioning and work of breathing [19,20,21], as well as cardiorespiratory regulation and prone and supine position during newborn sleep [22].

There are also publications on the recommendations of spending time in ventral decubitus when the baby is awake and its relationship with cranial deformities such as positional plagiocephaly [23,24], on the motor determinants in the motor development of the first months of life [25], and the relationship of abnormal primitive reflexes with the acquisition of motor milestones [26]. More specifically, the analysis of baby posture and the development of motor patterns has been studied by means of clinical assessment scales; specifically, there is a greater volume of publications that study posture in premature infants [27]. However, there are no publications that study posture in babies without pathology, which is relevant because it could lead to the achievement of normal motor parameters that would facilitate the detection of possible alterations during the first months of life. The study by Gajewska et al. [25] examines how motor elements observed at 3 months of age may influence motor performance at 6 months. It focuses on the relationship between early motor skills and the subsequent development of motor skills at a later age, for example, in dorsal decubitus, the most prominent motor pattern is the turning from dorsal to ventral decubitus position around the sixth month of life, which positively influences the development of physiological and appropriate spinal curvatures. Other conclusions of this study were that, in ventral decubitus, the position of the scapula and pelvis in the third month of life had the greatest impact on achieving adequate support of the upper extremities in the sixth month of life. On the other hand, the position of the pelvis and lower extremities in the third month of life in dorsal decubitus influenced all the characteristics observed in ventral decubitus at the age of 6 months.

Therefore, research on baby posture is essential to assess the motor development of babies, as well as to identify potential problems that may require early intervention.

## 5. Limitations

This study has several limitations, which are detailed below:

- This study considers postural control in a short period of time after placing the baby on the platform. Future studies, with a more robust postural analysis tool, could analyze a longer period of time in which the child develops different motor activities.
- Only the motor area is assessed; the rest of the developmental areas, such as the emotional, sensory, manipulative, communicative, and social areas, are not assessed. The development of postural control is the first thing that becomes evident, but it is also conditioned by the rest of the areas. Given the age of our study sample, this is difficult to analyze, but in subsequent studies with older children, it might be interesting to determine the relationships between these skills and postural control.
- One of the major drawbacks in early detection is the difficulty that clinicians have in determining whether a child’s postural control or motor skills can be classified within normo-typical development or whether they belong to abnormal development or motor delay with respect to the baby’s chronological or corrected age (in the case of premature babies). Normally, these differences become evident with time, but during the first months of life, there are many difficulties in determining these changes. However, once a valid analytical tool is developed, a study could be conducted with a larger sample, including healthy and pathological children of different ages, to determine whether it is possible to diagnose motor disorders in infants earlier.

## 6. Conclusions

Early detection of delays in motor or postural development is necessary to optimize effective treatment, and new early detection methods, such as deep learning techniques, are needed; however, it is necessary to know the area of support of babies with normo-typical development.

The development of a predictive tool for motor delay will allow defining the area of support of each baby according to its age range. The design of a prototype capable of detecting support in babies will reveal the postural control of these children at an early age.

The DL model will facilitate decision-making by health professionals. If the model can correctly detect the infant’s areas of support, it will be able to learn what the correct postural control is at each stage of the baby’s first months. It will be possible to determine which support areas are normal and which are not, and, above all, whether there is more support surface than is appropriate for the baby’s age. This is very important because it is difficult for human eyesight to be precise, and subjectivity comes into play. However, with a postural analysis tool, decision-making will be more precise and objective.

Having the prototype will make it possible to know the degree of deviation from normality, being able to suspect any abnormality, and allowing the corresponding health professional to make a clinical judgment or diagnosis if necessary, reducing the time it takes to be detected with the crucial advantages that this entails.

## 7. Patents

The results of the present study have been protected under the utility model with registration number U202432052.

## Data Availability

Data supporting the findings of this study are available from the corresponding author upon reasonable request. The data are not publicly available due to privacy.

## Abbreviations

The following abbreviations are used in this manuscript:

AI	Artificial intelligence
CNN	Convolutional Neural Network
DL	Deep learning
GPI	Graphics Processing Unit
